# Semaphorin 3A promotes activation of Pax7, Myf5, and MyoD through inhibition of emerin expression in activated satellite cells

**DOI:** 10.1002/2211-5463.12050

**Published:** 2016-04-27

**Authors:** Mulan Qahar, Yuko Takuma, Wataru Mizunoya, Ryuichi Tatsumi, Yoshihide Ikeuchi, Mako Nakamura

**Affiliations:** ^1^ Department of Animal and Marine Bioresource SciencesGraduate School of AgricultureKyushu UniversityHakozakiFukuokaJapan

**Keywords:** activated satellite cells, emerin, Myf5, MyoD, Pax7, Sema3A

## Abstract

We previously showed that Semaphorin 3A (Sema3A) expression was induced when quiescent muscle satellite cells were stimulated by hepatocyte growth factor and became activated satellite cells (ASCs). However, how Sema3A regulates genes in the early phase of ASCs remains unclear. In this study, we investigated whether Sema3A signaling can regulate the early phase of ASCs, an important satellite cell stage for postnatal growth, repair, and maintenance of skeletal muscle. We showed that expression of the myogenic proliferation regulatory factors Pax7 and Myf5 was decreased in myoblasts transfected with Sema3A siRNA. These cells failed to activate expression MyoD, another myogenic proliferation regulatory factor, during differentiation. Interestingly, some of the Sema3A‐depleted cells did not express Pax7 and MyoD and had enlarged nuclei and very large cytoplasmic areas. We also observed that Pax7 and Myf5 expression was increased in Myc‐Sema3A overexpressing myoblasts. BrdU analysis indicated that Sema3A regulated proliferation of ASCs. These findings suggest that Sema3A signaling can modulate expression of Pax7, Myf5, and MyoD. Moreover, we found that expression of emerin, an inner nuclear membrane protein, was regulated by Sema3A signaling. Emerin was identified by positional cloning as the gene responsible for the X‐linked form of Emery–Dreifuss muscular dystrophy (X‐EDMD). In conclusion, our results support a role for Sema3A in maintaining ASCs through regulation, via emerin, of Pax7, Myf5, and MyoD expression.

**Abbreviations**
ASC: activated satellite cell
DM: differentiation medium
EDL: extensor digitorum longus
EGF: epidermal growth factor
FGF2: basic fibroblast growth factor
GM: growth medium
HGF: hepatocyte growth factor
Pax7: paired box gene 7
QSC: quiescent satellite cell
Sema3A: semaphorin 3A
X‐EDMD: X‐linked Emery Dreifuss muscular dystrophy

Muscle regeneration is initiated by activation of muscle stem cells, known as satellite cells, which are adjacent to the basal lamina around the proximal region of each myofiber 1, 2, 3, 4. Satellite cells are in a quiescent state until they become activated by external stimuli triggered by muscle injury 4, 5, 6. Once the satellite cell is activated, quiescent satellite cells (QSCs) are transiently changed to an active state and are then called active satellite cells (ASCs). ASCs function as myogenic progenitor cells that can be differentiated into myotubes 2, 3, 4. Proliferation of ASCs is also essential to maintain the number of stem cells that can then regain characteristics of QSCs 4, 7, 8.

Pax7 was shown to be a key factor in understanding the contradictory QSC and ASC functions of satellite stem cells. Pax7 is expressed in the QSCs and remains expressed in ASCs when differentiation markers Myf5 and MyoD have been activated 2, 4, 8. Pax7 expression is gradually downregulated as differentiation proceeds toward formation of matured muscle fibers 2, 4, 8. Decreasing Pax7 expression was reported to promote skeletal muscle differentiation 9, 10. This suggests that maintenance of Pax7 expression is crucial to the switch from the ASC state to the initiation of myocyte differentiation. Pax7 expression levels in ASC are regulated in a differentiation‐dependent manner 8. Muscle tissues of adult conditional Pax7 KO mice had normal structure, indicating that muscle had formed normally, but the tissues showed a significant loss of muscle regeneration capacity, attributed to the loss of QSCs 11, 12.

Semaphorin 3A (Sema3A) is a secreted protein first reported as playing a role in neuronal repulsive signaling. Semaphorin 3A and its receptors, neuropilin and plexin, were shown to guide axons during formation of the nervous system. Subsequently, it was revealed that Sema3A was involved not only in neurogenesis but also in various physiological events such as angiogenesis and the immune response 13, 14. We previously showed that stimulating QSCs with EGF or HGF induced Sema3A expression prior to myotube formation 15 and Sema3A enhanced myogenic differentiation potential both *in vitro* and *in vivo* 15, 16. This indicated that a stepwise mechanism converted self‐renewing ASCs to differentiated ASCs, even though cells at both stages expressed Pax7. Our present study focused on investigating the genes involved in the change from self‐renewing to differentiated ASCs.

To address Sema3A regulation in the transition from self‐renewing to differentiated ASCs, we hypothesized existence of feedback regulation between Sema3A and Pax7 expression. Here, we demonstrated that inhibition of Sema3A expression impaired that of Pax7 and Myf5 and resulted in increased expression of emerin, an early differentiation marker that appears prior to MyoD expression. These data suggest that Sema3A might function to maintain Pax7 and Myf5 expression for commitment to myogenesis.

## Materials and methods

### Cell culture

Satellite cell‐derived myoblasts were isolated from skeletal muscle harvested from C57B/6J mice, which were a generous gift from K. Ojima, Institute of Livestock and Grassland Science, National Agriculture and Food Research Organization (Tsukuba, Ibaraki, Japan). For clonal culture, myoblasts were maintained in the following growth medium (GM): Ham's F10 Nutrient Mixture medium (F10; Invitrogen, Grand Island, NY, USA) with 20% fetal bovine serum (FBS; Invitrogen), 1% antibiotic–antimycotic mixture (Invitrogen), 0.5% gentamicin (Invitrogen) and 2.5 ng·mL^−1^ recombinant rat fibroblast growth factor‐2 (FGF‐2, R&D Systems, Minneapolis, MN, USA) at 37 °C. To induce differentiation, myoblasts were incubated with the following differentiation media (DM): Dulbecco's Modified Eagle Medium (DMEM with high glucose, Invitrogen) with 5% horse serum (HS; Invitrogen), 1% antibiotic–antimycotic mixture, and 0.5% gentamicin.

### siRNA transfection

Myoblasts were transfected with 100 nm Sema3A siRNA (Invitrogen) or control siRNA (Invitrogen) using Dharma FECT transfection reagents (Thermo Scientific, Waltham, MA, USA) according to the manufacturer's instructions. siRNA target sequences were: control, 5′ AAGCCGGTATGCCGGTTAAGT 3′; Sema3A, 5′ GGACATCATCCTGAGGACAACATTT 3′.

### Plasmid DNA transfection

Full‐length mouse Sema3A cDNA fragments were PCR‐amplified and subcloned into a myc‐tagged pCS2MT vector. The fragment including five myc tags were excised with BamHI and Xho/blunted sites and subcloned into a pIRES2‐EGFP+MT vector (Clontech Laboratories, Inc., San Jose, CA, USA) at BglII and SmaI sites. The pIRES2‐EGFP+MT‐Sema3A (Sema3A vector) or empty vector were transiently transfected into myoblasts using TransITR‐LT1 transfection reagents (Mirus Bio LLC, Madison, WI, USA) according to the manufacturer's instructions.

### Reverse transcription‐polymerase chain reaction

Total RNA was isolated from cultured myoblasts using RNeasy Micro kit (Qiagen, Hilden, Germany) according to the manufacturer's protocol. cDNA was synthesized from total RNA by a reverse transcriptase SuperScript III (Invitrogen) using an oligo(dT) primer. mRNA expression of Sema3A, Pax7, Myf5, and MyoD were monitored by real‐time quantitative PCR (RT‐qPCR) using Roche LightCycler1.5 (Mannheim, Germany) run under the TaqMan probe detection format standardized with hypoxanthine guanine phosphoribosyl transferase (HPRT). The primer sets were designed using the probefinder (version 2.35 for mouse; Roche) with an intron‐spanning assay for mouse Sema3A, Pax7, Myf5, and MyoD, as shown in Table 1. Annealing temperature was set to 60 °C in all cases.

**Table 1:** PCR primer sets for mouse Sema3A, Pax7, Myf5, MyoD, and HPRT

Primer	Accession number	Left primer	Right primer
Sema3A	NM_009152.4	atcagtgggtgccttaccaa	gccaaatgttttactgggaca
Pax7	NM_011039.2	ggcacagaggaccaagctc	gcacgccggttactgaac
Myf5	NM_008656.5	ctgctctgagcccaccag	gacagggctgttacattcagg
MyoD	NM_010866.2	agcactacagtggcgactca	ggccgctgtaatccatca
HPRT	NM_013556.2	cctcctcagaccgcttttt	aacctggttcatcatcgctaa

### Preparation of conditioned media for ECL western blot analysis

Cells were passaged and transfected with Sema3A vector or empty vector as described above. After transfection, the conditioned media were collected and each sample filtered through a 0.2‐μm filter to ensure removal of any dead cells and mixed with the same volume of 2× sample buffer for SDS/PAGE. After boiling for 5 min, samples were concentrated using spin columns with a MW cutoff of 3000 Da (Vivaspin, Sartorius, Goettingen, Germany). The concentrated samples were analyzed by western blotting 15.

### Whole cell extracts and ECL western blotting

Whole cell extracts were harvested in 1× SDS/PAGE sample buffer, electrophoresed on 10% polyacrylamide gels under reducing conditions and transferred to nitrocellulose membranes as described previously 15, 17. The membranes were blocked with 5% skim milk in 0.1% polyethylene sorbitan monolaurate (Tween 20)‐Tris buffered saline (T‐TBS) for 1 h at room temperature, followed by incubation overnight at 4 °C in a 1 : 1000 dilution of primary antibodies against Sema3A (polyclonal; Abcam, Cambridge, UK), Pax7 (generously provided by Y. Ohkawa, Kyushu University, Japan), Myf5 (polyclonal; Santa Cruz Biotechnology, Inc. Santa Cruz, CA, USA), MyoD (polyclonal; Santa Cruz Biotechnology. Inc.), MyHC (monoclonal; R&D Systems Inc.), emerin (polyclonal; Santa Cruz Biotechnology, Inc.), Lmo7 (polyclonal; Santa Cruz Biotechnology, Inc.) or β‐actin (monoclonal; Abcam). Antibodies were diluted in CanGetSignal solution 1 (Toyobo, Osaka, Japan) containing 0.05% NaN_3_. The membranes were washed three times (10 min each) with T‐TBS and further incubated with a 1 : 5000 dilution of biotinylated goat anti‐rabbit IgG (Vector Laboratories, Burlingame, CA, USA), biotinylated rabbit anti‐goat IgG (Vector Laboratories), HRP‐conjugated anti‐mouse IgG (DAKO, Tokyo, Japan) or HRP‐conjugated anti‐rabbit IgG (DAKO) secondary antibodies in CanGetSignal solution 2 (Toyobo) for 1 h at 25 °C. The membranes were washed as above and then those that had been incubated with biotinylated secondary antibodies were further incubated in horseradish peroxidase (HRPO)‐labeled avidin (Vector Laboratories) at a 1 : 500 dilution in T‐TBS for 30 min. The membranes were washed as above and HRP activity was detected using an enhanced chemiluminescence (ECL) detection kit (GE Healthcare, Little Chalfont, UK) read in a Fusion SL4 system (M&S Instruments Trading Inc., Osaka, Japan). Band intensities of immunoblots were assessed using imagej software (National Institute of Health, Bethesda, MD, USA).

### Immunocytochemistry

Myoblasts were fixed in 4% paraformaldehyde (PFA) for 20 min then steamed for 20 min in 10 mm citrate buffer. They were then washed three times in PBS and incubated in 0.2% Triton X‐100 (Thermo Fisher Scientific, Fair Lawn, NJ, USA) in PBS for 20 min to permeabilize the cells. Subsequently, cells were treated for 1 h with a blocking buffer consisting of 3% BSA, 5% goat serum in T‐TBS. Primary antibody solutions containing rabbit polyclonal antibody against Sema3A (1 : 200; Millipore, Billerica, MA, USA) and mouse monoclonal antibody against Pax7 (1 : 200, generously provided by Y. Ohkawa, Kyushu University) and rabbit polyclonal antibody against MyoD (1 : 200, also from Ohkawa) were incubated with the cells overnight at 4 °C. Cells were then washed three times with T‐TBS and incubated with secondary antibodies containing Alexa‐Fluor‐488‐conjugated goat anti‐rabbit IgG (1 : 500; Invitrogen), Alexa‐Fluor‐594‐conjugated goat anti‐mouse IgG (1 : 500; Invitrogen), or DAPI (1 : 1000) for 1 h at room temperature. All antibodies were diluted in 3% BSA in T‐TBS. For positive cell number evaluation, six microscopic fields were randomly selected from each group, at 20‐fold magnification, and the numbers of Pax7+/MyoD+, Pax7+/MyoD−, Pax7−/MyoD+, and Pax7−/MyoD− nuclei were calculated in each field using imagej software. This experiment was performed on both Sema3A and control siRNA transfected cells.

### BrdU incorporation assay

Cells transfected with either siRNA or plasmid DNA were grown in a 24‐well plate and pulse‐labeled with 10 μm BrdU (Sigma‐Aldrich, St. Louis, MO, USA) for 2 h. Cells were fixed in 100% methanol with 0.1% H_2_O_2_ for 10 min at 4 °C, incubated in 2 N HCI for 1 h at 37 °C and neutralized with 1× TBE buffer (pH 8.5). Subsequently, cells were incubated overnight at 4 °C with G3G4 anti‐BrdU monoclonal antibody (1 : 100 dilution in 0.1% BSA‐PBS; The Developmental Studies Hybridoma Bank, Iowa City, IA, USA). Cells were then washed and incubated with HRP‐conjugated anti‐mouse IgG antibody (1 : 500 dilution in 0.1% BSA‐PBS, Sigma) for 2 h at room temperature. BrdU‐positive cells were visualized by staining with 3,3′‐diaminobenzidine (DAB; Sigma‐Aldrich, 1 mg·mL^−1^ DAB and 0.02% H_2_O_2_ in PBS) 18, 19.

## Results

### Suppression of Sema3A expression resulted in decreased Pax7 and Myf5 levels

**Figure 1:**
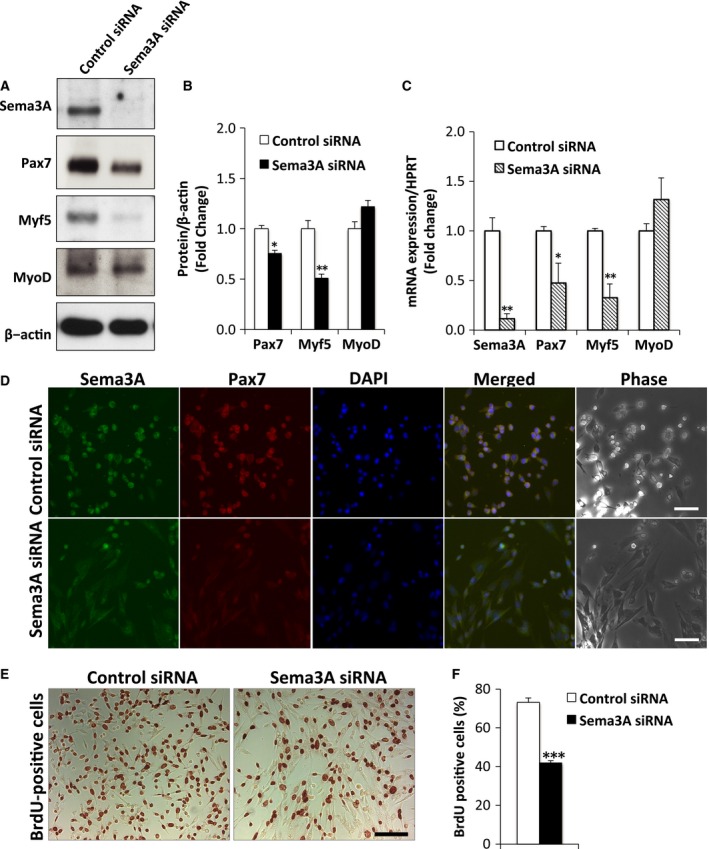
Suppression of Sema3A expression resulted in decreased Pax7 and Myf5 levels. (A) Myoblasts were transfected with Sema3A or control siRNA. After 2 days of transfection in GM, cells were lysed and lysates analyzed by western blotting for protein expression of Sema3A, Pax7, Myf5, and MyoD. β‐actin was used as an internal control. (B) Representative western blots showing expression of Pax7, Myf5, and MyoD in myoblasts transfected with Sema3A or control siRNA (A). The results are plotted as values relative to β‐actin expression. Data are means ± S.E. *P < 0.05, **P < 0.01 vs. control siRNA. (C) Myoblasts were transfected with Sema3A or control siRNA. After 2 days of transfection in GM, total RNA was extracted and RT‐qPCR was performed using primers specific for Sema3A, Pax7, Myf5, and MyoD. The mRNA expression of each gene was normalized to that of HPRT and plotted relative to the expression of each transcript. The data are means ± S.E. *P < 0.05, **P < 0.01 vs. control siRNA. (D) Immunofluorescence staining of Sema3A (green) or Pax7 (red) in myoblasts after treatment with Sema3A or control siRNA for 48 h. Nuclei were counterstained with DAPI (blue). Bar, 150 μm. (E) BrdU‐staining (brown) in myoblasts after treatment with Sema3A or control siRNA for 48 h. Bar, 100 μm. (F) Percentage of BrdU‐positive nuclei in myoblasts after treatment with Sema3A or control siRNA for 48 h; *n* = 16 per group. Data are means ± S.E. ***P < 0.01 vs. control siRNA.

To address the function of Sema3A in myogenic cells, we performed Sema3A knockdown in cultured myoblasts derived from satellite cells of skeletal muscle from C57BL/6J mice and evaluated expression of myogenic transcription factors to assess myogenic potential. Sema3A or control siRNAs were transfected into cells and total protein and RNA were extracted 2 days after transfection. Immunoblotting revealed that Sema3A knockdown resulted in markedly decreased levels of Pax7 and Myf5 proteins without affecting MyoD levels (Fig. 1A). Protein expression of Myf5 and Pax7 was decreased 24% and 49%, respectively (Fig. 1B). No significant changes in MyoD protein levels were detected in Sema3A siRNA transfected cells, compared with control cells (Fig. 1B). We further evaluated expression of these transcription factors by RT‐qPCR. In cells transfected with Sema3A siRNA, Sema3A mRNA levels were suppressed by 90% compared with in control cells (Fig. 1C). The mRNA expression of Pax7 and Myf5 was decreased by 60% and 80%, respectively (Fig. 1C). However, there was no significant difference in MyoD mRNA levels between cells treated with Sema3A and those treated with control siRNAs (Fig. 1C). To determine if the observed decreases in Pax7 and Myf5 expression occurred in a specific population of cells, we performed immunocytochemistry utilizing Sema3A and Pax7 antibodies. Compared with the control myoblasts, decreased Pax7 expression in myoblasts with Sema3A knockdown was substantial and was homogenously distributed among all cells in the field (Fig. 1D). The immunocytochemistry results confirmed that inhibition of Sema3A expression caused decreased Pax7 expression in most cells. These data indicated that Sema3A functions to maintain Pax7 and Myf5 transcription but does not affect MyoD expression. In addition, recent studies demonstrated that Pax7 regulated Myf5 expression by binding directly to its promoter and activating its expression 20. This previous report supports the hypothesis that Sema3A regulates expression of Myf5 via Pax7. It was reported that Pax7‐mutant satellite cells did not proliferate because of cell cycle arrest 21, 22. Furthermore, *in vitro* studies confirmed that Pax7 promoted proliferation of satellite cells 8. On the basis of this, we performed a BrdU assay to test whether Sema3A would affect satellite cell proliferation. The BrdU analysis showed that Sema3A depletion led to decreased cell proliferation. The Sema3A siRNA treated cells had significantly fewer BrdU‐positive cells than the controls (Fig. 1E,F). Taken together, our results demonstrated that Sema3A might be required for maintenance of ASCs.

### Sema3A knockdown decreased MyoD expression during early stage of differentiation

**Figure 2:**
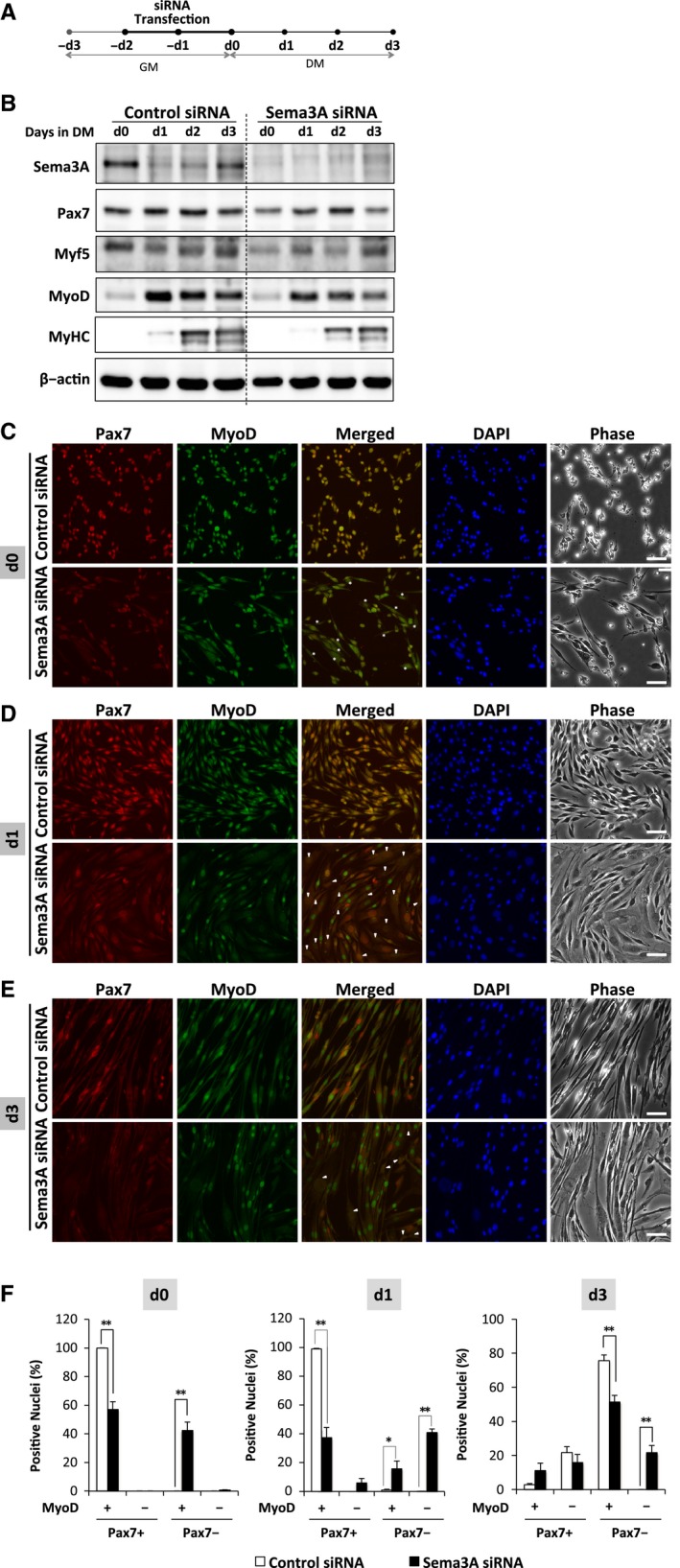
Downregulation of Sema3A reduced expression of myogenic markers during myoblast differentiation. (A) Schematic diagram of the experimental time schedule. Myoblasts were transfected with Sema3A or control siRNA. After 2 days of transfection in GM (from day**−**2 to day 0), the medium was changed to DM for 3 days (day 0 to day 3). Samples were collected every day for 3 days and subjected to western blotting analysis. (B) Protein expression of Sema3A, Pax7, Myf5, and MyoD was monitored throughout differentiation in myoblasts transfected with Sema3A or control siRNA. β‐actin was used as an internal control. Control siRNA (left), Sema3A siRNA (right). (C–E) Immunofluorescence staining for Pax7 (red) or MyoD (green) in myoblasts after treatment with Sema3A or control siRNA at d0, d1, and d3. Nuclei were counterstained with DAPI (blue). Bar, 50 μm; Asterisk, Pax7−/MyoD+ cells; Arrowhead, Pax7−/MyoD− cells. (F) Percentages of Pax7+/MyoD+, Pax7+/MyoD−, Pax7−/MyoD+, Pax7−/MyoD− nuclei in control and Sema3A siRNA transfected cells at d0, d1, and d3. Data are means ± S.E. *P < 0.05, **P < 0.01 vs. control siRNA.

**Figure 3:**
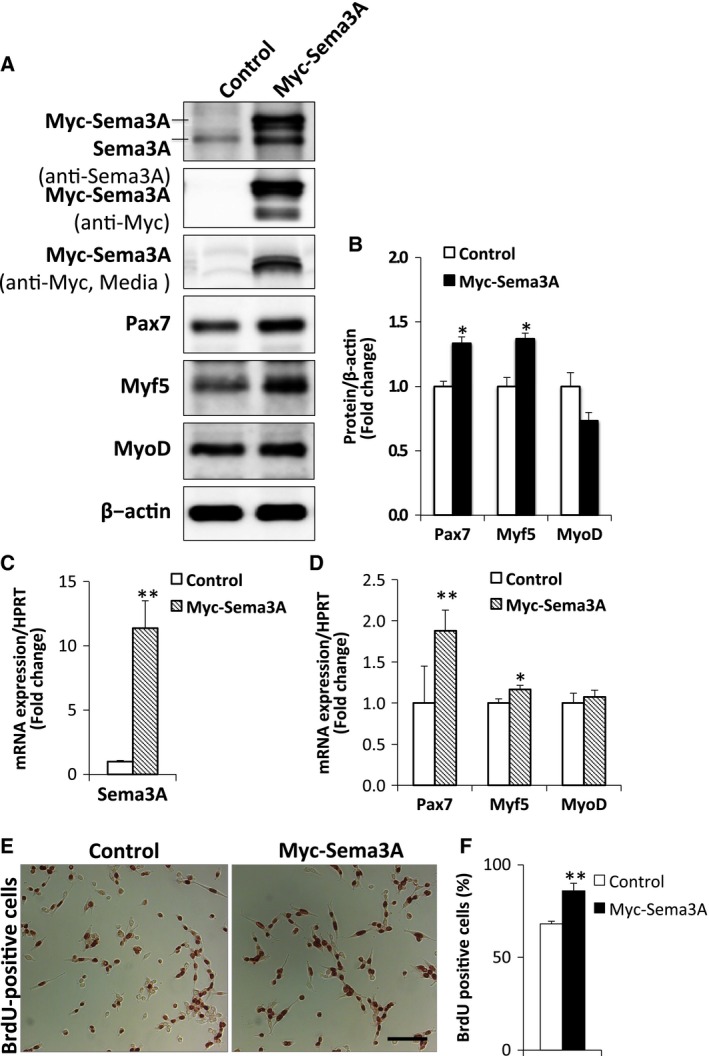
Overexpression of Sema3A increased Pax7 and Myf5 protein expression. (A) Myoblasts were transfected with Myc‐Sema3A or control vector and, after 2 days of transfection in GM, cell lysates and conditioned medium from the each culture were extracted and analyzed by western blotting. Protein expression of endogenous Sema3A and Myc‐Sema3A were detected in cell lysates using Sema3A and Myc antibodies, respectively. Myc‐Sema3A in conditioned media was determined using the Myc antibody. Pax7, Myf5, and MyoD proteins were detected in cell lysates using the corresponding antibodies. β‐actin was used as an internal control. (B) Representative western blots showing protein expression of Pax7, Myf5, and MyoD in myoblasts transfected with Myc‐Sema3A or control vector (A). The results are expressed as values relative to β‐actin expression. Data are means ± S.E. *P < 0.05, **P < 0.01 vs. control vector. (C, D) Myoblasts were transfected with Myc‐Sema3A or control vector. After 2 days of transfection in GM, total RNA was extracted and RT‐qPCR performed using primers specific for Sema3A, Pax7, Myf5, and MyoD. The mRNA expression of each gene was normalized by that of HPRT and plotted relative to the expression of each transcript. The data are means ± S.E. *P < 0.05, **P < 0.01 vs. control vector. (E) BrdU‐staining (brown) in myoblasts after treatment with Myc‐Sema3A or control vector for 48 h. Bar, 100 μm. (F) Percentage of BrdU‐positive nuclei in myoblasts after treatment with Myc‐Sema3A or control vector for 48 h; *n* = 16 per group. **P < 0.01.

To test the hypothesis that Sema3A is required for ASC maintenance, we evaluated the differentiation potential of cells after siRNA transfection (Fig. 2A). Myoblasts were transfected with Sema3A or control siRNA in GM for 2 days and the medium was changed to DM, with incubation for another 3 days. A late myogenic differentiation marker, myosin heavy chain (MyHC) appeared beginning at d1 and its expression increased during differentiation of the control cells (Fig. 2B), indicating successful myogenic differentiation. However, in Sema3A siRNA transfected cells, MyHC expression was suppressed (Fig. 2B). In cells transfected with control siRNA, Sema3A was expressed at d0 but decreased upon induction of differentiation (Fig. 2B). In Sema3A siRNA transfected cells, there was no Sema3A expression throughout the differentiation period (Fig. 2B). Pax7 and Myf5 expression was decreased at d0 in cells with Sema3A knockdown, confirming our other findings (Fig. 1) and their expression remained low until d3 (Fig. 2B). MyoD expression was low in both control and Sema3A siRNA transfected cells at d0, confirming that the cells are early stage ASCs. Although MyoD expression was not affected at d0, its expression was induced in control cells once the medium was changed to DM. MyoD expression was low from d1 to d3 in Sema3A siRNA transfected cells, probably because of low Pax7 and Myf5 expression. Immunofluorescence staining showed that the control cells had nuclear expression of Pax7 and MyoD at d0. Pax7 expression was decreased in Sema3A depleted cells (Fig. 2D) and it is consistent with the prior immunohistochemistry (Fig. 1D). Staining for MyoD at d0 was diffuse in Sema3A siRNA transfected cells but was not substantially lower than in control cells. This was consistent with western blotting analysis (Fig. 1A). Culturing cells in DM for 1 days resulted in clear differences in MyoD expression (Fig. 2D). DM induced MyoD expression in the control cells, as confirmed by western blotting analysis (Fig. 2B). This induction was impaired in the Sema3A siRNA transfected cells. Quantitative analysis showed that, with Sema3A siRNA transfection, the percentage of proliferating cells (Pax7+/MyoD+) was only 37%, while it was over 98% with control siRNA (Fig. 2D,F). In addition, of Sema3A siRNA transfected cells, 15% were differentiating (Pax7−/MyoD+) and 5.9% were self‐renewing (Pax7+/MyoD−) (Fig. 2D,F). Interestingly, with Sema3A siRNA transfection, about 40% of Pax7−/MyoD− cells had enlarged nuclei and very large cytoplasmic areas (Fig. 2D,F). These features were never observed in control cells. The cells started fusing at d3 and the percentage of Pax7−/MyoD+ nuclei in Sema3A siRNA transfected cells were significantly lower than that in control cells (Fig. 3E,F), indicating less myogenic differentiation. In the control and Sema3A siRNA transfected cells, there were 21% and 15% self‐renewing cells (Pax7+/MyoD−), respectively (Fig. 2E,F). The Pax7−/MyoD− cells were also observed at d3 and only with Sema3A siRNA transfection (Fig. 3E,F). In previous reports, cells in a senescent state had enlarged nuclei and very large cytoplasmic areas 23, 24. Further research should address the possibility that, in this cell fate transition, Pax7−/MyoD− cells can cease differentiating and enter a senescent state.

### Overexpression of Sema3A increased Pax7 and Myf5 expression

As downregulation of Sema3A reduced expression of the myogenic markers Pax7 and Myf5 (Figs 1 and 2), we next investigated whether overexpression of Sema3A would upregulate Pax7 and Myf5 expression. To test this, we transfected a myc‐tagged‐Sema3A‐pIRES2‐EGFP expression vector (Myc‐Sema3A) or pIRES2‐EGFP control vector (Control) in myoblasts. The efficiency and specificity of overexpression was confirmed by measuring Myc‐Sema3A protein in whole cell lysates by western blotting with Myc and Sema3A antibodies (Fig. 3A). To test whether transfected Myc‐Sema3A was secreted into the medium, we performed western blotting with a Myc antibody, detecting Myc‐Sema3A in the conditioned medium from Myc‐Sema3A transfected myoblasts (Fig. 3A). Next, we tested protein expression of Pax7, Myf5 and MyoD by western blotting with the corresponding antibodies. As expected, protein levels of Pax7 and Myf5 were increased, by 1.3‐ and 1.4‐fold, respectively, in Sema3A‐overexpressing myoblasts (Fig 3A,B). However, there was no significant change in MyoD protein levels in cells transfected with the Myc‐Sema3A vector, as compared with in control cells (Fig 3A,B). We further evaluated expression of these transcription factors by RT‐qPCR. In cells transfected with the Myc‐Sema3A vector, compared with in control cells, Sema3A mRNA expression was increased 11.4‐fold (Fig. 3C), and Pax7 and Myf5 mRNA by 1.9‐ and 1.7‐fold, respectively (Fig. 3D). Although, there was no significant difference between MyoD mRNA levels in Myc‐Sema3A vector transfected and control cells (Fig. 3D). We next performed a BrdU assay to determine whether overexpression of Sema3A increased satellite cell proliferation. Sema3A overexpressing myoblasts had more BrdU‐positive nuclei than did controls (Fig. 3E,F). Taken together, our results indicated that Sema3A signaling plays a critical role in maintenance of ASCs by regulation of Pax7 and Myf5.

### Sema3A regulated protein expression of emerin

**Figure 4:**
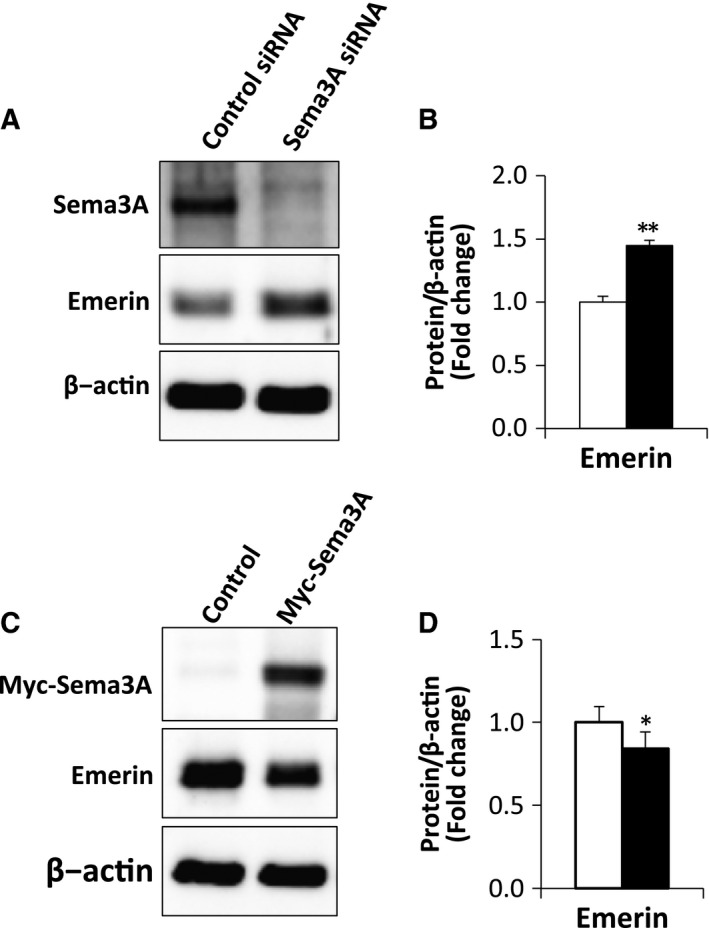
Sema3A regulated protein expression of emerin. (A) Myoblasts were transfected with Sema3A or control siRNA. After 2 days of transfection in GM, cells were lysed and lysates analyzed by western blotting for protein expression of Sema3A and emerin. β‐actin was used as an internal control. (B) Representative western blots showing emerin protein expression in myoblasts transfected with Sema3A or control siRNA (A). The results are expressed as values relative to β‐actin expression. Data are means ± S.E. *P < 0.05, **P < 0.01 vs. control siRNA. (C) Myoblasts were transfected with Myc‐Sema3A or control vector. After 2 days of transfection in GM, cells were lysed and subjected to western blotting. Protein expression of Myc‐Sema3A and emerin were identified using Myc and emerin antibodies, respectively. β‐actin was used as an internal control. (D) Representative western blots showing protein expression of emerin in myoblasts transfected with Myc‐Sema3A or control vector (Fig. 3A). Results are expressed as values relative to those of β‐actin expression. The data are means ± S.E. *P < 0.05, **P < 0.01 vs. control vector.

Emerin is an inner nuclear membrane protein that was identified by positional cloning as the gene responsible for the X‐linked form of Emery–Dreifuss muscular dystrophy (EDMD) 25. Emerin was shown to inhibit expression of myogenic regulatory factors, including Pax7 and Myf5 26. Furthermore, Myf5 was increased in myoblasts from emerin‐null mice 27. On the basis of these reports, we investigated whether or not emerin expression in myoblasts was regulated by Sema3A signaling. We first confirmed that Sema3A siRNA‐induced knockdown in myoblasts affected emerin expression. As expected, emerin protein expression was increased 1.5‐fold in myoblasts with Sema3A knockdown (Fig. 4A,B). Next, we investigated emerin expression in Sema3A overexpressing myoblasts (Fig. 3A). We found that upregulation of Sema3A caused a 16% decrease in emerin expression (Fig 4C,D). Taken together, these results suggested that emerin expression was negatively regulated by Sema3A signaling.

## Discussion

We previously reported that Sema3A was expressed in early phase ASCs and that its expression was induced by HGF and EGF 15. However, how Sema3A is involved in early phase ASCs has remained unclear. In this study, we showed that the loss of Sema3A led to decreased expression of Pax7, a representative satellite cell marker. It also decreased expression of Myf5, another ASC marker that is co‐expressed with Pax7. We found that Myf5 expression was decreased at both the RNA and protein levels by Sema3A depletion. In contrast, cells overexpressing Sema3A, as compared with control transfected cells, showed increased expression of Pax7 and Myf5. This suggested that Sema3A could induce Pax7 and Myf5 expression to keep satellite cells in the early ASC stage.

Measuring Pax7, Myf5, and MyoD protein levels is an accepted method of monitoring the status of satellite cells 2, 4, 8. The QSCs (Pax7+/Myf5−/MyoD−) progress toward differentiation in a stepwise manner once they are activated 2, 4, 8. Myf5 expression is initiated at the early stage of ASC (Pax7+/Myf5+/MyoD−), then MyoD expression is activated (Pax7+/Myf5+/MyoD+) 2, 4, 8. Pax7 and Myf5 expression disappears before the cells enter the late ASC stage (Pax7**−**/Myf5**−**/MyoD+) 2, 4, 8. The goal of this study was to show whether Sema3A could drive transition from the early stage of ASCs into the ASCs. We confirmed that Sema3A promoted myoblast proliferation and this could be explained by increased Pax7 and Myf5 expression (Figs 1A–E and 3A–F). We also found that neither knockdown nor overexpression of Sema3A affected MyoD expression in myoblasts that were still in a growth condition (Fig 1A–C and 2B,C). However, the Sema3A‐induced decreases in Pax7 and Myf5 levels led to lower expression of MyoD once the cells were in differentiation medium (Fig. 2B). Decreased Pax7 and Myf5 levels resulted in lower expression of MyoD during differentiation, consistent with reports of MyoD as a downstream marker of these two genes 28, 29. In cells treated with Pax7 siRNA, MyoD expression during differentiation was suppressed (data not shown), confirming regulation of MyoD expression by Pax7. Therefore, we propose that Sema3A‐induced Pax7 and Myf5 can upregulate MyoD activation to drive transition from the early stage of ASC into the ASC stage. Interestingly, we observed that, in Sema3A siRNA transfected cells in DM, Pax7−/MyoD− cells had enlarged nuclei and very large cytoplasmic areas (Fig. 2D,E). It was reported that myogenic cells in a senescent state are no longer proliferative and their nuclei and cytoplasmic areas were enlarged 23, 24. Therefore, we propose that Sema3A‐knockdown induced some of the cells into a senescent state. Further analysis, such as staining those cells with senescence markers, would be needed to address this question. Furthermore, our results showed that myoblast differentiation was inhibited by Sema3A siRNA transfection. The western blotting results showed that expression of the myogenic differentiation marker MyHC was suppressed in Sema3A siRNA transfected cells in DM (Fig. 2B). We also found fewer Pax7−/MyoD+ nuclei in Sema3A siRNA transfected cells at d3 (Fig. 2E,F). It is reasonable to postulate that, in different cells, transfection with Sema3A siRNA could result in different cell fates, that is, either differentiated, self‐renewing or senescent phenotypes. Taken together, our findings suggest that Sema3A precisely regulates the ASC state of myogenic cells.

**Figure 5:**
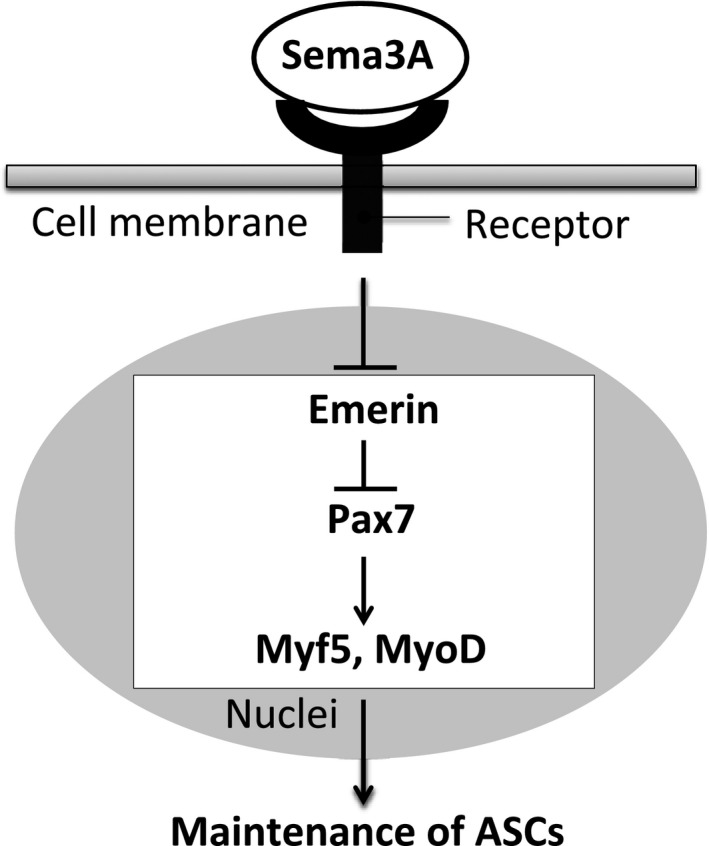
Model for Sema3A‐induced ASCs maintenance. In myoblasts, Sema3A signaling inhibits emerin expression via its receptor to promote activation of Pax7, Myf5 and MyoD. This coupling may contribute to maintenance of ASCs.

Sema3A is a secreted factor 30 and the transfected Myc‐Sema3A protein was found in the culture medium when overexpressed (Fig. 3D). We also confirmed that the myoblasts without any transfection could secrete endogenous Sema3A into the medium (data not shown). Neuropilin‐1 and plexin‐A2 bind and form the known receptor for the Sema3A ligand. We previously reported that neuropilin‐1 and plexin‐A2 were expressed in satellite cells in EDL and soleus muscles 16. However, the signal transduction processes in these cells remains unclear. One remaining question is how Sema3A regulates Pax7, Myf5 and MyoD expression. Sema3A is known to induce osteoblast differentiation through activating β‐catenin signaling 31. Interestingly, β‐catenin binds to emerin and its function is increased in emerin‐null cells 32. Expression of emerin and Pax7 in QSCs and ASCs was confirmed in muscle fiber cultures 33. Emerin inhibited Pax7 and Myf5 expression 26 and myoblasts from emerin‐null mice had increased Myf5 expression 27. It was, in addition, reported that emerin‐null myoblasts from X‐EDMD patients were more proliferative 34. Our findings and these reports indicated that Sema3A could regulate Pax7 and Myf5 through a pathway dependent on both β‐catenin and emerin. Compared with control cells, Sema3A knockdown upregulated emerin expression, while Sema3A overexpressing cells had reduced emerin expression (Fig. 4A–D). These findings support the hypothesis that Sema3A signaling negatively regulates emerin expression and, furthermore, promotes Pax7 and Myf5 activation through emerin inhibition (Fig. 5). This is the first report to address the relationship among Sema3A, emerin, Pax7, and Myf5. Mutation or loss of emerin causes X‐EDMD and functional defects in emerin affect gene expression and cell signaling 35. Our data showed that Sema3A overexpression elevated Pax7 and Myf5 expression and repressed that of emerin (Fig. 4C,D). This suggests that the loss of emerin would impair the balance between proliferation and differentiation normally regulated by Sema3A. Loss of emerin might mimic the state of myoblasts in X‐EDMD patients. Though expression levels of Sema3A, Pax7, and Myf5 have not been reported in X‐EDMD patients, more research on these factors and emerin signaling *in vivo* as well as *in vitro* would build an understanding of the mechanism and function of satellite cell differentiation and potentially advance clinical applications for X‐EDMD.

## Author contribution

MQ, RT, and MN conceived and designed the project. MQ and YT performed the experiments and acquired the data. RT, WM, YI and MN contributed conceptual insights. MQ and MN wrote the paper.
